# Seagrass meadows mixed with calcareous algae have higher plant productivity and sedimentary blue carbon storage

**DOI:** 10.1002/ece3.8579

**Published:** 2022-02-14

**Authors:** Olivia J. Kalokora, Martin Gullström, Amelia S. Buriyo, Matern S. P. Mtolera, Mats Björk

**Affiliations:** ^1^ Dar es Salaam University College of Education (DUCE) Dar es Salaam Tanzania; ^2^ School of Natural Sciences, Technology and Environmental Studies Södertörn University Huddinge Sweden; ^3^ 107660 Department of Botany University of Dar es Salaam Dar es Salaam Tanzania; ^4^ 107660 Institute of Marine Sciences University of Dar es Salaam Zanzibar Tanzania; ^5^ 7675 Department of Ecology, Environment and Plant Sciences Stockholm University Stockholm Sweden

**Keywords:** blue carbon, calcification, *Halimeda opuntia*, plant interactions, productivity, seagrass meadows, *Thalassia hemprichii*

## Abstract

Seagrass meadows capture and store large amounts of carbon in the sediment beneath, thereby serving as efficient sinks of atmospheric CO_2_. Carbon sequestration levels may however differ greatly among meadows depending on, among other factors, the plant community composition. Tropical seagrass meadows are often intermixed with macroalgae, many of which are calcareous, which may compete with seagrass for nutrients, light, and space. While the photosynthetic CO_2_ uptake by both seagrasses and calcareous algae may increase the overall calcification in the system (by increasing the calcium carbonate saturation state, Ω), the calcification process of calcareous algae may lead to a release of CO_2_, thereby affecting both productivity and calcification, and eventually also the meadows’ carbon storage. This study estimated how plant productivity, CaCO_3_ production, and sediment carbon levels were affected by plant community composition (seagrass and calcareous algae) in a tropical seagrass‐dominated embayment (Zanzibar, Tanzania). Overall, the patterns of variability in productivity differed between the plant types, with net areal biomass productivity being highest in meadows containing both seagrass and calcareous algae. Low and moderate densities of calcareous algae enhanced seagrass biomass growth, while the presence of seagrass reduced the productivity of calcareous algae but increased their CaCO_3_ content. Sedimentary carbon levels were highest when seagrasses were mixed with low or moderate cover of calcareous algae. The findings show that plant community composition can be an important driver for ecosystem productivity and blue carbon sequestration.

## Graphical abstract

This study estimated how plant productivity, CaCO_3_ production, and sediment carbon levels were affected by plant community composition (seagrass and calcareous algae). Sedimentary carbon levels were highest when seagrasses were mixed with low or intermediate coverage of calcareous algae. The findings show that plant community composition can be an important driver for ecosystem productivity and blue carbon sequestration.

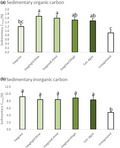

## 1 INTRODUCTION

Seagrass meadows are ecologically and economically important habitats globally and recognized for provision of numerous highly valuable ecosystem goods and services (Costanza et al., 1997; Unsworth & Cullen, 2010). In recent years, they have been increasingly acknowledged as major carbon sinks contributing up to 15% of the total carbon stored in the ocean (Kennedy & Björk, 2009). Their efficiency in carbon storage is attributed to high metabolic rates of seagrass (Duarte et al., 2010) producing excess carbohydrate stored in the underground parts (Duarte & Cebrian, 1996), which together with an efficient filtering capacity results in a high capture of both autochthonous and allochthonous carbon into the sediment (Fourqurean, Duarte, et al., 2012). However, seagrass meadows, especially in the tropics, are commonly mixed with calcareous algae, such as *Halimeda* spp. and *Penicillus* spp. (Kangwe et al., 2012; Ortegón‐Aznar et al., 2017). Interactions between seagrass and calcareous algae may influence community development (Schoener, 1983), productivity (Barry et al., 2013; Davis & Fourqurean, 2001), and thus potentially also sediment carbon sequestration. The strength and nature of such processes may however differ depending on the proportion of a meadow’s plant components, which has not yet been studied in detail.

Calcareous algae have been shown to contribute greatly to the CaCO_3_ fraction of tropical seagrass sediments, for example, in the Western Indian Ocean (Muzuka et al., 2001) and Caribbean (Gischler, 1994; Ortegón‐Aznar et al., 2017). Nevertheless, calcareous algae may also increase seagrass productivity by causing release of CO_2_ by their calcification. Calcium carbonate (CaCO_3_) precipitation reduces seawater pH and might thereby be causing a release of CO_2_ (Frankignoulle et al., 1994, 1995; Ware et al., 1992), as recently shown in calcareous algae (Kalokora et al., 2020), even though the photosynthetic uptake of CO_2_ may exceed that of the release from calcification (Cornwall et al., 2018). The CO_2_ released from calcification can either escape to the atmosphere, thereby contributing to increases in atmospheric CO_2_, or be utilized by the calcareous algae themselves and nearby plants through photosynthesis (Invers et al., 2001; Schneider & Erez, 2006; Semesi et al., 2009), leading to greater productivity and higher rates of blue carbon sequestration (Russell et al., 2013). At the same time, photosynthetic CO_2_ uptake by seagrasses increases the calcium carbonate saturation state, Ω, and pH of the surrounding seawater, which can enhance the calcification of both calcareous algae (Semesi et al., 2009) and corals (Unsworth et al., 2012) within the habitats, thereby also affecting the amount of CO_2_ released from calcification (Björk & Beer, 2009; Macreadie et al., 2017). Calcareous algae may therefore largely influence the carbon cycle in seagrass meadows and hence affect their carbon storage.

In this study, we conducted a field survey to assess how plant productivity, calcium carbonate production, and sedimentary carbon levels were affected by interaction between a seagrass, *Thalassia hemprichii* (Ehrenberg) Ascherson, and calcareous algae (*Halimeda* spp., mostly *H*. *opuntia* Lamouroux) at different relative cover in a tropical bay of Zanzibar, Tanzania. Specifically, we tested the following hypotheses: (i) an increase in relative cover of seagrass would reduce the primary productivity of the calcareous algae due to shading; (ii) an increase in relative cover of calcareous algae would increase the primary productivity of seagrass due to increased availability of CO_2_ from calcification; (iii) calcification of calcareous algae will increase with increased relative cover of seagrass, even though shading might to some extent reduce primary productivity; and (iv) a higher relative cover of seagrass will result in higher organic carbon storage in the sediment, whereas a higher cover of calcareous algae would result in a higher inorganic carbon storage in the sediment.

## 2 MATERIALS AND METHODS

### 2.1 Study area and field sampling design

**FIGURE 1:**
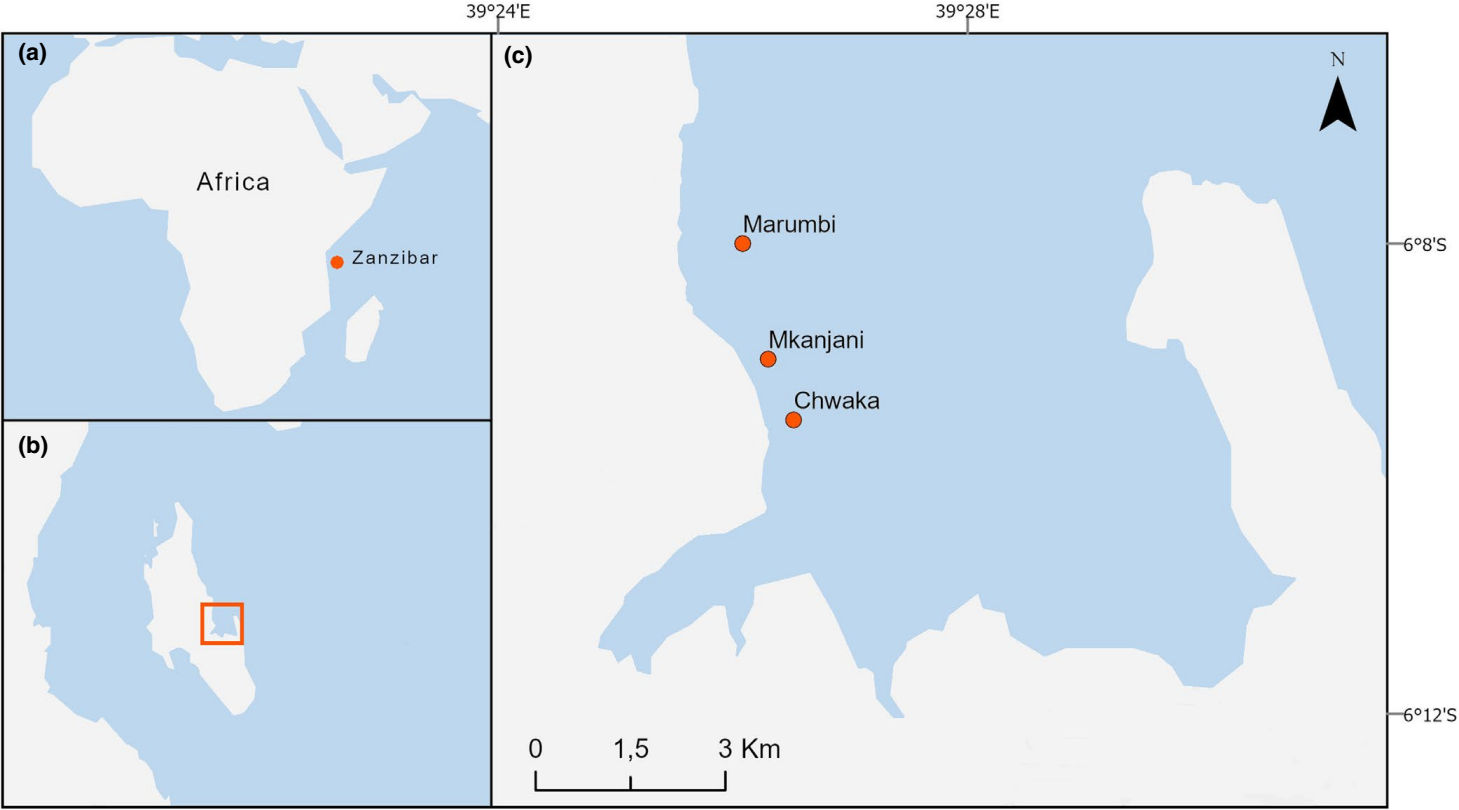
Map showing the general study area and the three specific study sites located in Chwaka Bay in Unguja Island, Zanzibar (Tanzania)

The field survey was conducted in Chwaka Bay on the east coast of Unguja Island, Zanzibar, Tanzania (6^o^8’ to 6^o^15’S, 39^o^22’ to 39^o^30’E). The bay is a semi‐enclosed embayment with a maximum average tidal range of 3.2 m at spring tide (Cederlöf et al., 1995). The bay is dominated by extensive seagrass meadows (of varying densities) and encompasses up to 11 species of seagrass (Gullström et al., 2012), which are commonly mixed with different species of the calcareous algal genus *Halimeda* (Gullström et al., 2006). The study was conducted from May to July 2017 during the South Eastern (SE) monsoon period, which is characterized by weak rainfall and low stable air temperatures (Mahongo, 2014). Within the bay, three representative sites (Chwaka [CenterPoint: 6°09’30"S, 39°26’31"E], Mkanjani [CenterPoint: 6°08’59"S, 39°26’18"E], and Marumbi [CenterPoint: 6°07’60"S, 39°26’05"E]) were chosen (Figure 1) containing meadows dominated by *Thalassia hemprichii* (a climax seagrass species widely distributed across the Indo‐pacific region; Short et al., 2007), and a large proportion of calcareous green algae (*Halimeda* spp., mostly *H*. *opuntia*) identified according to Oliveira et al. (2005). To describe the general vegetation patterns of the three sites, we determined structural habitat characteristics (seagrass shoot density, thalli density of calcareous algae, and plant height; Table S1) using conventional methodologies described by Duarte et al. (2001). General estimations of physicochemical parameters and light were made for each site. During three days, at morning, midday, and evening (*n* = 3 at each time), dissolved oxygen, salinity (conductivity), and temperature were measured using a YSI Multimeter (YSI 85/10 Ohio USA), pH was estimated using a Hanna Combo meter (HI98129 Hanna Instruments USA) and light intensity was measured using a quantum meter (Li‐Cor model LI‐189 Japan) (Table S2).

The three studied sites contained three randomly selected plots (15 m^2^) each (located 4–5 m apart), where comparisons have been done on 4 or 5 subplots (0.25 × 0.25 m) of different plant community compositions. Each subplot was hence replicated 9 times (3 sites × 3 plots). In total, there were 70 subplots, including 15 response variables with 4 subplots and 2 response variables with 5 subplots (Table 1, Table S3). Plant community compositions were divided into the following categories:

1. Seagrass (S): 90%–100% cover of *T*. *hemprichii*
2. S_high_CA_low_: High cover (70%–80%) of *T*. *hemprichii*, low cover (20%–30%) of *H*. *opuntia*
3. S_mid_CA_mid_: Moderate cover (45%–55%) of *T*. *hemprichii*, moderate cover (45%–55%) of *H*. *opuntia*
4. S_low_CA_high_: Low cover (20%–30%) of *T*. *hemprichii*, high cover (70%–80%) of *H*. *opuntia*
5. Calcareous algae (CA): 90%–100% cover of *H*. *opuntia*.

**TABLE 1:** Plant metrics used as response variables in univariate comparisons (Figures 2, 3, and Table S3) and correlative assessments (Figure 4) of plant community composition categories

Plant category	Abbreviation
Response variable	
Seagrass	
Seagrass shoot biomass productivity (Equation 1) (g DW day^−1^ shoot^−1^)	SeagShoBioProd
Seagrass leaf elongation rate per leaf (Equation 2) (mm day^−1^ leaf^−1^)	SeagLeafElongLeaf
Seagrass leaf elongation rate per shoot (Equation 3) (mm day^−1^ shoot^−1^)	SeagLeafElongSho
Seagrass leaf production per shoot (Equation 4) (number of leaves day^−1^ shoot^−1^)	SeagLeafProSho
Seagrass net areal shoot biomass productivity (Equation 5) (g DW m^−2^ day^−1^)	SeagAreaBioPro
Seagrass coverage^a^ (% cover of seagrass)	SeagCov
Calcareous algae	
Calc algae thalli weight (g DW thallus^−1^)	CABiom
Calc algae segment growth (Equation 6)^a^ (segments day^−1^ tip^−1^)	CASegmGro
Calc algae biomass productivity (Equation 7) (g DW day^−1^ thalli^−1^)	CABioPro
Calc algae net areal biomass productivity (Equation 8)^a^ (g DW m^−2^ day^−1^)	CAAreaBioPro
Calc algae CaCO_3_ in mature thalli segments (Equation 9)^a^ (% DW CaCO_3_)	CACaCO3Mat
Calc algae CaCO_3_ in new thalli segments (Equation 9)^a^ (% DW CaCO_3_)	CACaCO3New
Calc algae CaCO_3_ in whole thalli (Equation 9)^a^ (% DW CaCO_3_)	CACaCO3Who
Calc algae CaCO_3_ productivity (Equation 10) (g CaCO_3_ day^−1^ thallus^−1^)	CACaCO3Pro
Calc algae net areal CaCO3 productivity (Equation 11) (g CaCO_3_ m^−2^ day^−1^)	CAAreaCaCO3Pro
Calc algae standing CaCO3 (Equation 12) (g CaCO_3_ m^−2^)	CACaCO3Stand
Calc algae coverage^a^ (% cover of calc algae)	CACov
Total net areal biomass productivity (Equation 5 + 8) (g DW m^−2^ day^−1^)	TotAreaBioPro

^a^ Response variables where data have been transformed due to heterogeneous variances (for details, see Data analysis).

The subplots were selected based on the natural distribution of the species, and no manipulations were made. All measurements were executed during low spring tide.

### 2.2 Measurements of growth and production of seagrass and calcareous algae

Growth of seagrass was determined by the hole punch technique (Short et al., 2001). In each subplot (except CA), 5–7 shoots of approximately similar size (c. 13–16 cm; Suppl. Table 1) were punched by piercing a needle through all blades in a shoot to make a hole just above the basal meristem region. The punched seagrass shoots were marked by colored cable tags for easy relocation during harvesting. Similarly, in each subplot (except Seag), 3–4 algal thalli of approximately the same size (c. 7 cm; Table S1) were selected and marked using cable tags without disturbing the plant (Ballesteros, 1991). The cable tags were placed on the top segment (usually the fifth segment), and during subsequent harvesting, the segments above the cable tag were counted as new segments and the rest were the mature segments. Marked seagrass shoots and algal thalli were left to grow in situ for 13 days after which they were harvested. In addition, three algal thalli adjacent to the tagged ones were collected from each plot (except for Seag) for determining the percent of CaCO_3_ per thalli. Harvested seagrass shoots were rinsed with freshwater, scraped to remove epiphytes, and rinsed again with water before being separated into new and mature parts by cutting the leaf at the hole. Seagrass leaves without punch marks were considered new leaves. Plant material was then oven dried at 60°C to constant weight. The amount of new plant material per time yielded seagrass shoot biomass productivity (g DW day^−1^ shoot^−1^, Equation 1), seagrass leaf elongation rate per leaf (mm day^−1^ leaf^−1^, Equation 2), seagrass leaf elongation rate per shoot (mm day^−1^ shoot^−1^, Equation 3), and seagrass leaf production per shoot (number of leaves day^−1^ shoot^−1^, Equation 4):

(1) $$\text{Seagrass shoot biomass productivity}=\frac{\text{New biomass growth per shoot}}{\text{Time}}$$

(2) $$\text{Seagrass leaf elongation rate per leaf}=\frac{\text{Leaf increment in a leaf}}{\;\text{Time}}$$

(3) $$\text{Seagrass leaf elongation rate per shoot}=\frac{\text{Leaf increment per all leaves in a shoot}}{\;\text{Time}}$$

(4) $$\text{Seagrass leaves produced per shoot}=\frac{\text{Number of new leaves formed per shoot}}{\text{Time}\;}$$

Seagrass net areal shoot (aboveground) biomass productivity (g DW m^−2^ day^−1^, Equation 5) was calculated by multiplying production per shoot by the appropriate mean shoot density per m^2^:

(5) Seagrass net areal shoot biomass productivity=Seagrass shoot biomass productivity x shoot density

For calcareous algae, marked thalli were separated into mature and new segments; rinsed with freshwater to remove sand, debris, and epiphytic material; and wrapped separately in aluminum foil before being oven dried at 60°C to constant weight. The growth of calcareous algae segments (segments day^−1^ tip^−1^, Equation 6) was calculated from the number of new segments per time, while the thalli biomass productivity (g DW day^−1^ thalli^−1^, Equation 7) was estimated from new biomass growth per time:

(6) $$\text{Calcareous algae segment growth}\;=\frac{\text{Number of new segments}\;}{\text{Time}}$$

(7) $$\text{Calcareous algae biomass productivity}\;=\frac{\text{Biomass growth per thalli}\;}{\text{time}}$$

Calcareous algae net areal biomass productivity (g DW m^−2^ day^−1^, Equation 8) was calculated by multiplying thalli biomass productivity with thalli density per m^2^:

(8) Calcareous algae net areal biomass productivity=Thalli biomass productivity x Thalli density

Finally, the total net areal biomass productivity (for vegetated subplots) was calculated by adding seagrass net areal shoot biomass productivity (Equation 5) to calcareous algae net areal biomass productivity (Equation 8).

### 2.3 Determination of CaCO_3_ content and calcification

Dried samples of new, mature, and whole thalli (i.e., three thalli apart from the marked ones collected from each plot during harvesting) were ground to a fine powder using mortar and pestle. CaCO_3_ content was estimated after acidification of the dried samples following Kennedy et al. (2005). Three subsamples of 2 g each were then taken from each sample and treated with excess 2 M HCl until the effervescence stopped. All samples were oven dried at 60°C to constant weight, left to cool, and then reweighed. The CaCO_3_ content of calcareous algae (in mature or new segments and in whole thalli) was calculated from the average weight loss (Equation 9):

(9) $${\text{Calcareous algae CaCO}}_{3}\;\text{in thalli segments}=\frac{\text{Initial weight before acidification}‐\text{Final weight after acidification}\;}{\;\text{Initial weight before acidification}}\times 100$$

The relative CaCO_3_ productivity was calculated as the increase in CaCO_3_ per thallus over time (g CaCO_3_ day^−1^ thallus^−1^, Equation 10), and the net areal CaCO_3_ productivity (g CaCO_3_ m^−2^ day^−1^, Equation 11) was calculated by multiplying the relative productivity with the thallus density per area:

(10) $${\text{Calcareous algae CaCO}}_{3}\;\text{productivity}=\frac{{\text{CaCO}}_{3}\;\text{increase per thallus}}{\text{Time}}$$

(11) $${\text{Calcareous algae net areal CaCO}}_{3}\;\text{productivity}={\;\text{CaCO}}_{3}\;\text{productivity}\times \text{thalli density}$$

The standing stock of CaCO_3_ in living calcareous algae (g CaCO_3_ m^−2^, Equation 12) was estimated by multiplying the content of CaCO_3_ in thalli with thallus density:

(12) $${\text{Calcareous algae standing CaCO}}_{3}={\text{CaCO}}_{3}\;\text{content per thalli}\;\times \text{thalli density}$$

### 2.4 Determination of organic and inorganic carbon content of the sediment

At the end of the survey, one sediment sample was taken from each subplot (*n* = 9) using a corer (8 cm in diameter) pushed down to 15 cm depth. In the vicinity of each plot (just outside the meadow but close to the plot), three sediment cores were taken from unvegetated sediments (*n* = 9). Larger shells, infauna, and large plant materials were removed from the sediment before being oven dried at 60°C to constant weight. Samples were homogenized by grinding using mortar and pestle and prewashed using ethanol to minimize contamination. The amount of organic carbon was determined by the Loss on Ignition (LOI) method described by Heiri et al. (2001). Following this method, each subsample (0.1 g) was dried at 90–100°C for one hour and allowed to cool at room temperature in a desiccator, reweighed to obtain dry weight before combustion, and then combusted in a muffle furnace (450°C for 16 h). Samples were allowed to cool at room temperature and reweighed to obtain dry weight after combustion. The percentage of organic matter loss (% LOI) was calculated according to Equation 13. Since % LOI includes other element such as nitrogen, oxygen, and sulfur, the percentage of organic carbon (% C_org_) was calculated using LOI as a proxy of C_org_ according to Fourqurean et al. (2012) using Equation 14. The percentage of inorganic carbon in the sediment was calculated from the weight difference between the pre‐ashed and weight loss during ignition as shown in Equation 15.

(13) $$\%\;\text{loss on ignition}\;\left(\text{LOI}\right)=\frac{\text{Dry weight before combustion}‐\text{Dry weight after combustion}}{\;\text{Dry weight before combustion}\;}\times 100$$

(14) %sediment organic carbon=0.43×%LOI‐0.33

(15) $$\%\;\text{sediment inorganic carbon}\;=\frac{\text{Dry weight before combustion}‐\text{Weight loss during combustion}}{\text{Dry weight before combustion}\;}\times 100$$

### 2.5 Data analysis

Data from the measurements of biomass, productivity, CaCO_3_ content, and calcification of the two plant types were analyzed using nested analysis of variance (ANOVA) with Site (3 levels) as a fixed factor and Plot (3 levels) as a random factor nested within Site. As the different response variables (Table 1) did not differ among the three sites, one‐way ANOVAs were performed to test for differences among the four or five subplot categories of plant community compositions of each response variable (*n* = 9). To limit the probability of Type I errors, adjustment of the significant level for multiple testing was performed following the Bonferroni procedure (Holm, 1979). Differences in sedimentary organic and inorganic carbon content among subplots were tested separately using one‐way ANOVA. Prior to all ANOVA analyses, homogeneity of variances was checked using Levene’s test. When the data were heteroscedastic, log_10_(x+1) transformations were performed. In those cases where data remained heteroscedastic despite transformation, the nonparametric Kruskal–Wallis test was used. Tukey’s test was used for pairwise comparisons between different subplot categories. The relative importance of a range of predictor variables on plant and CaCO_3_ productivity was assessed using partial least squares (PLS) regression analysis on Log_10_‐transformed data using SIMCA 13.0.3 software (UNETRICS, Malmö, Sweden). PLS regression analysis comprises a multivariate statistical technique (based on covariances), where predictive models are constructed from multiple response variables and multiple explanatory (predictor) variables. This method allows for identifying underlying factors by modeling of linear (or polynomial) relationships between one or several response variables (i.e., Y from a matrix) and predictors (i.e., X from a matrix) known as latent variables. The relative importance of the selected predictors is assessed by modeling projections to latent structures, that is, variables with the best predictive power. Q^2^ statistics is based on a matrix of regression coefficients and applied to measure the cross‐validated variance and hence predictability of different models (with a significant level of 5%). R^2^y cum is a measure of the cumulative fraction of selected predictors and provides a measure of model fit to the original data. In contrast to many other regression methods, PLS regression modeling is a useful statistical technique when the number of independent variables is large, and one must deal with multi‐collinearity (Carrascal et al., 2009). It has been used in different types of ecological research (Asplund et al., 2011; Gullström et al., 2018; Staveley et al., 2017).

## 3 RESULTS

### 3.1 Plant productivity, CaCO_3_ content, and calcification

**FIGURE 2:**
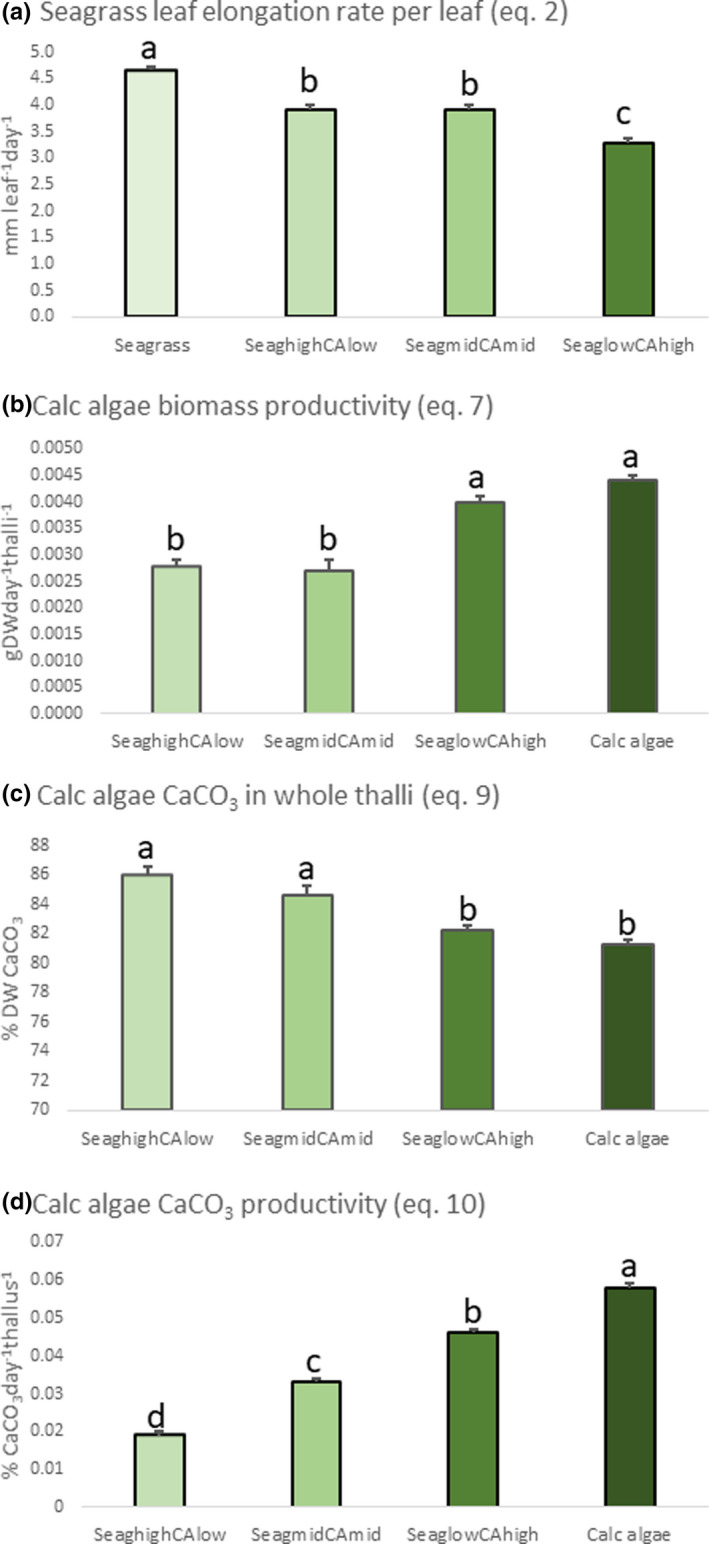
Plant (seagrass [Seag] and calcareous [calc, CA] algae) productivity as well as CaCO_3_ productivity and content (mean ± SE) measured in the different subplots (*n* = 9). (a) Seagrass leaf elongation rate per leaf, (b) Calc algae biomass productivity, (c) Calc algae CaCO_3_ in whole thalli, and (d) Calc algae CaCO_3_ productivity. Letters above bars indicate significant differences (based on results from Tukey’s post hoc tests) between different subplots for each response variable

**FIGURE 3:**
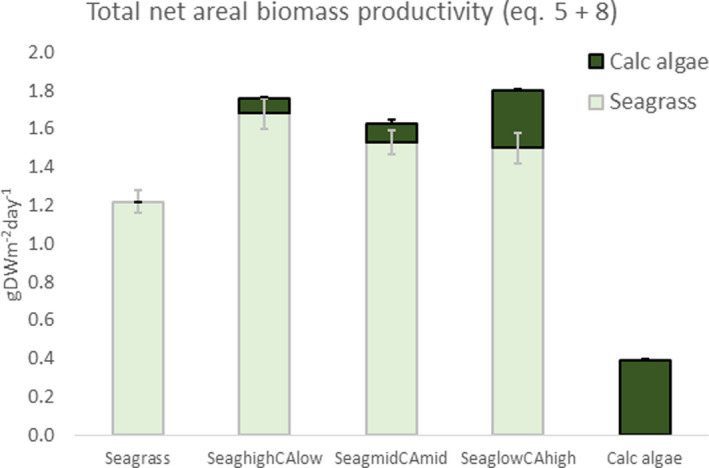
Total net areal biomass productivity as the sum of seagrass (Equation 5) and calcareous algae (Equation 8) (mean ± SE, *n* = 9)

**FIGURE 4:**
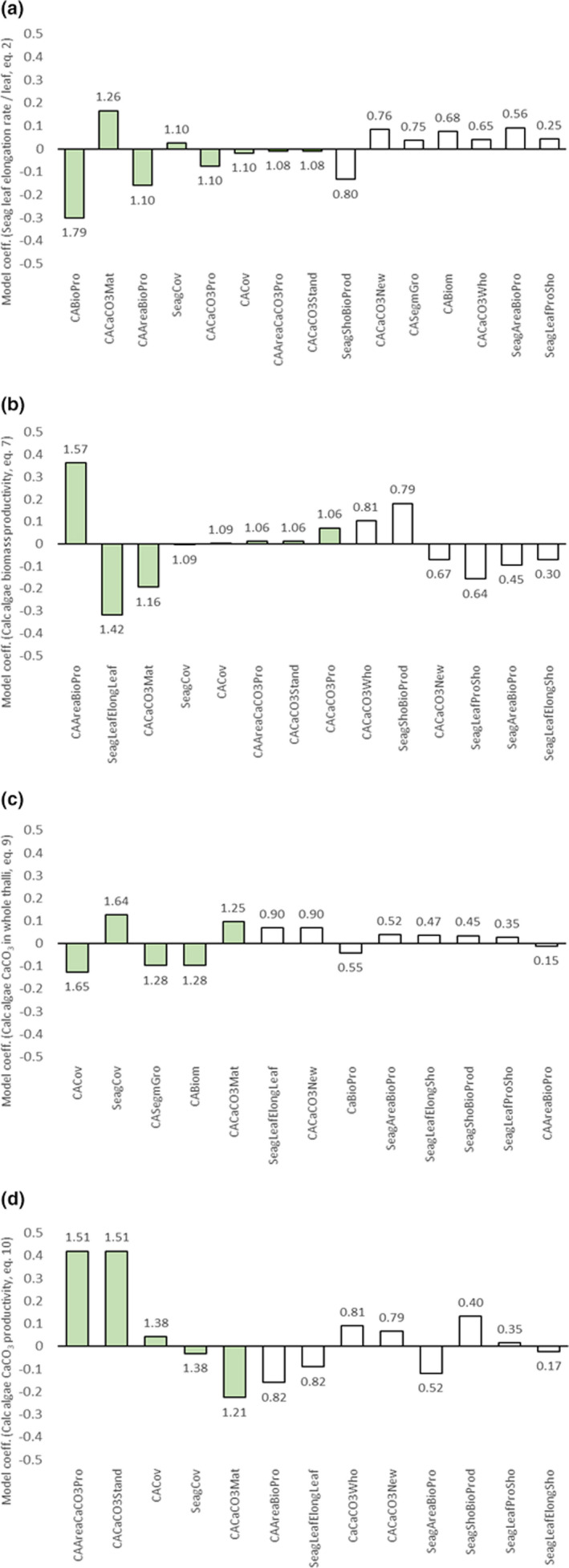
Coefficient plots of partial least square (PLS) regression models ranking (from left to right) the influence of seagrass and calcareous algae variables on (a) *seagrass leaf elongation rate per leaf* (mm day^−1^ leaf^−1^, Equation 2), (b) *calcareous algae biomass productivity* (g DW day^−1^ thalli^−1^, Equation 7), (c) *calcareous algae CaCO_3_ in whole thalli* (% DW CaCO_3_, Equation 9), and (D) *calcareous algae CaCO_3_ productivity* (g CaCO_3_ day^−1^ thallus^−1^, Equation 10). Green bars represent predictor variables with VIP (variable influence on the projection) values above 1, which include variables with an above average influence on the response variable in focus. White bars represent variables contributing less than average to the overall model of a certain response variable. The numbers above bars represent VIP values. Abbreviations are as follows: SeagShoBioProd (Seagrass shoot biomass productivity), SeagLeafElongLeaf (Seagrass leaf elongation rate per leaf), SeagLeafElongsho (Seagrass leaf elongation rate per shoot), SeagLeafProSho (Seagrass leaves produced per shoot), SeagAreaBioPro (Seagrass net areal shoot biomass productivity), SeagCov (seagrass coverage), CABiom (Calc algae thalli weight), CASegmGro (Calc algae segment growth), CABioPro (Calc algae biomass productivity), CAAreaBioPro (Calc algae net areal biomass productivity), CACaCO_3_Mat (Calc algae CaCO_3_ in mature thalli segments), CACaCO_3_New (Calc algae CaCO_3_ in new thalli segments), CACaCO_3_Who (Calc algae CaCO_3_ in whole thalli), CACaCO_3_Pro (Calc algae CaCO_3_ productivity), CAAreaCaCO_3_Pro (Calc algae net areal CaCO_3_ productivity), CACaCO_3_Stand (Calc algae standing CaCO_3_), and CACov (Calc area coverage). Noteworthy is that predictor variables that partly contain the same data as a response variable have been excluded from the analysis

All differences among subplots are described in detail in Figures 2, 3, 4 and Table S3, and below, we present general trends. Seagrass shoot biomass productivity (Equation 1) was lower in the unmixed seagrass subplot compared to all other subplots, while seagrass leaf elongation rate per leaf (Equation 2) increased with decreasing cover of calcareous algae. Seagrass leaf elongation rate per shoot (Equation 3) showed no clear trend, and we found no significant differences in the number of seagrass leaves produced per shoot (Equation 4).

The thalli weight (CABiom), segment growth (Equation 6), and biomass productivity (Equation 7) of calcareous algae were all higher when the seagrass cover was reduced. The CaCO_3_ content in thalli segments (i.e., mature segments, new segments, and whole thalli; Equation 9) was higher in meadows of higher seagrass cover. The CaCO_3_ productivity per thallus (Equation 10) was clearly higher in subplots of less seagrass cover.

The standing CaCO_3_ stock (Equation 12), cover of plants (SeagCov and CACov), and net areal productivity (Equations 5, 8, and 11) varied as a consequence of plant community composition and are given as background information and for further calculations. The total net areal biomass productivity ((5), (8) and (5), (8), Figure 3) was highest when seagrass was mixed with calcareous algae, followed by the seagrass subplots, which were in turn higher than the subplots containing only calcareous algae.

### 3.2 The relative importance of predictor variables on plant and CaCO_3_ productivity

Among the plant and CaCO_3_ productivity measures presented in Table 1, we selected the most relevant variables showing significant differences among subplots and general trends of interest (i.e., seagrass leaf elongation rate per leaf, calcareous algae biomass productivity, calcareous algae CaCO_3_ content in whole thalli, and calcareous algae CaCO_3_ productivity). All four PLS models indicated significant relationships between independent variables (predictors) and the measured (response) variables. The cross‐validated variance (Q^2^ statistics) ranged from 28 to 87% (i.e., higher than the significant limit level of 5%), and therefore, all models showed high predictability. The cumulative fraction of all the examined predictors combined (R^2^y cum) explained between 37 and 68% of the variation in the models, thereby displaying a relatively high degree of determination and fit.

Seagrass leaf elongation rate per leaf (Equation 2) was overall negatively correlated to productivity and cover of calcareous algae (biomass productivity, net areal biomass productivity, CaCO_3_ productivity, and cover of calcareous algae), while positively correlated with CaCO_3_ content in mature algal segments and seagrass cover (Figure 4a). Calcareous algae biomass productivity (Equation 7) showed positive relationships with net areal biomass productivity, cover, and CaCO_3_ productivity of calcareous algae and negative relationships with seagrass leaf elongation rate per leaf, CaCO_3_ content in mature algal segments, and seagrass cover (Figure 4b). Calcareous algae CaCO_3_ content in whole thalli (Equation 9) was positively correlated with seagrass cover and CaCO_3_ content in mature algal segments, whereas negatively correlated with cover, segment growth, and biomass of calcareous algae (Figure 4c). Calcareous algae CaCO_3_ productivity (Equation 10) was positively related to net areal productivity, CaCO_3_ standing stock, and cover of calcareous algae, and negatively related to seagrass cover and CaCO_3_ content of mature algal segments (Figure 4d).

### 3.3 Effects of plant cover on sedimentary blue carbon content

**FIGURE 5:**
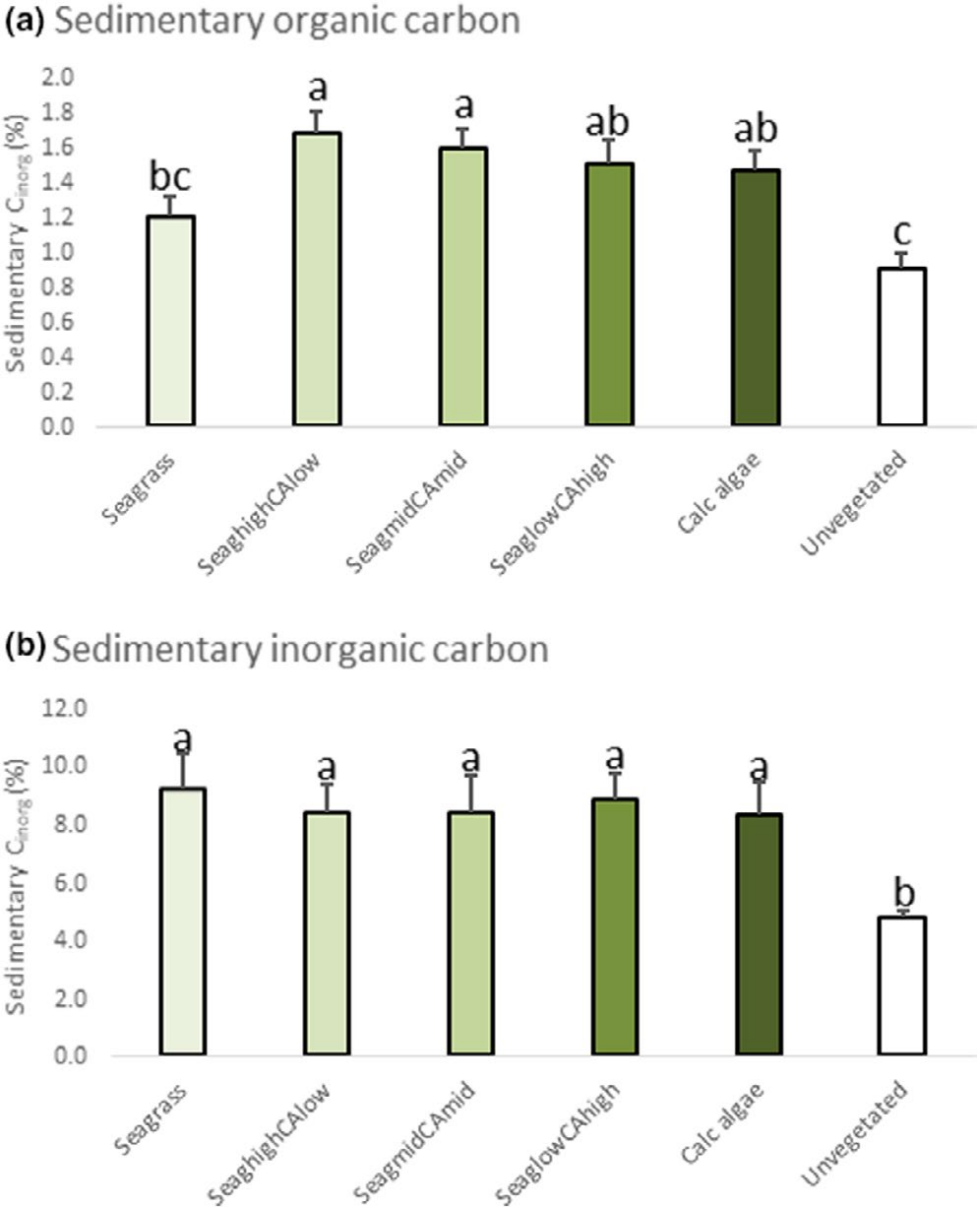
Sedimentary organic carbon (C_org_, a) and inorganic carbon (C_inorg_, b) content (%) in the different survey subplots with distinguished relative plant cover content (for details about proportion levels, see section “Study area and field sampling design” above) of sediment (0–15 cm depth). Bars show mean ±SE (*n* = 9)

Sediment organic carbon content was higher in vegetated than in the unvegetated sediment (Tukey’s test at *p* < .05), although this was not significant for the seagrass subplots (Figure 5a). The mixed subplots with high (Seag‐high/CA‐low) or mid seagrass content (Seag‐mid/CA‐mid) showed significantly higher content of organic carbon compared to non‐mixed subplots (Tukey’s test at *p* < .05, Figure 5a). For the inorganic carbon content of the sediment, the vegetated subplots showed clearly higher levels than the unvegetated sediment (Tukey’s test at *p* < .05, Figure 5b). No differences were found between any of the vegetated subplots (Figure 5b).

## 4 DISCUSSION

Both plant productivity and sedimentary carbon levels were higher in the mixed plant communities than in areas where plants were growing in monospecific stands. Overall, the productivity measures show that plant community composition generally has a lower influence on seagrass productivity than on productivity of calcareous algae. As hypothesized, an increased relative cover of seagrass had a significant negative effect on calcareous algal biomass productivity, probably as an effect of shading from seagrass leaves on algal thalli in high seagrass shoot densities (McCoy & Kamenos, 2015) and possibly also from competition for CO_2_ (Björk et al., 2004) or nutrients (Multer, 1988; South, 1983; Williams, 1990) that could result in large decreases in algal thalli size and biomass growth (Davis & Fourqurean, 2001). Although an increase in the relative cover of calcareous algae reduced the seagrass leaf elongation rate (also shown in the PLS analysis), the seagrass biomass production increased (as the “seagrass shoot biomass productivity” was enhanced) when the two plant types were growing together. Thus, seagrass leaves appear to become shorter and thicker in a seagrass meadow mixed with calcareous macroalgae and longer and thinner in dense seagrass stands, probably due to competition for light in plant communities with high seagrass shoot densities (Ralph et al., 2007). Thus, the observed changes in leaf elongation rate are not a measure of productivity, rather a morphological adaptation to the changes in ambient light availability.

Contrary to our expectations and based on both among‐subplot testing and PLS modeling, CaCO_3_ productivity of calcareous algae was shown to strongly negatively correlate with seagrass cover (thereby positively correlate with cover of calcareous algae). However, the proportion of CaCO_3_ in algal thalli increased in subplots with higher relative cover of seagrass, while thalli in monospecific subplots and subplots with higher relative cover of calcareous algae had lower CaCO_3_ content. A similar increase in CaCO_3_ content has been reported for *Halimeda incrassata* in seagrass meadows of the Cayman Islands (Barry et al., 2013). Such an increase in CaCO_3_ content might be explained by the effect of seagrass photosynthesis, which in these shallow, dense seagrass meadows drastically increases pH and Ω_aragonite_, which in turn can enhance macroalgal calcification (Anthony et al., 2011; Semesi et al., 2009). As a consequence, thalli of calcareous algae growing more scattered within a seagrass meadow will have a higher carbonate content, and possibly also be more resistant to herbivory as a high CaCO_3_ content has been suggested to enhance the effect of secondary metabolites that deter herbivores (Hay et al., 1994).

A positive correlation between sedimentary organic carbon and seagrass biomass has been reported from seagrass meadows in the western Indian Ocean (Gullström et al., 2018). Thus, we expected that the organic carbon content of the sediment would be highest in monospecific seagrass areas. It was however found that subplots with high or mid cover of seagrass mixed with calcareous algae had a significantly higher organic carbon content than the unmixed seagrass subplot. The presence of calcareous algae apparently enhanced sediment carbon storage even though the algae by themselves had a comparatively low organic carbon production. It is possible that the combination of different plant types creates a physically complex structure that will increase particulate organic matter trapping capacity, leading to an increase in the amount of organic carbon in the sediment. Structural complexity in seagrass meadows has been shown to affect organic matter sedimentation (Dahl et al., 2018), and complex macroalgal assemblages trap considerable amounts of sediments (Stamski & Field, 2006). It has also been found that in Tangkhen Bay, Thailand, *Halimeda* spp. plays an important role as a source of organic matter in sediment (Tuntiprapas et al., 2019).

Seagrass meadows generally contain a range of different vegetation community compositions depending on, for example, bottom characteristics, water quality, light penetration, and temperature (Björk et al., 2008). Calcareous algae are particularly common in tropical seagrass meadows (Ortegón‐Aznar et al., 2017), where they can influence the development of meadow configuration and plant composition (Schoener, 1983). In Chwaka Bay, mixed plant community compositions (that was found to benefit blue carbon stock levels) are commonly observed. Here, the relative cover of seagrass and *Halimeda* varies greatly within and among meadows, while there is no significant seasonal variation in general cover for either of the two plants studied here (Gullström et al., 2006). In the present study, the sedimentary organic content of seagrass meadows was dependent on the proportion of the meadow’s plant components. It is thus important to know how stable the relative plant cover mixtures are in space and time. While no data that show distribution patterns of seagrass versus *Halimeda* vegetation cover at a microscale (below meter scale) is available, the long‐time stability of the general vegetation composition reported from Chwaka Bay (Gullström et al., 2006) makes it probable that the reported vegetation mixtures assessed here are indeed persistent enough to have an impact on the productivity and sedimentary carbon content of the system.

The inorganic carbon content of the sediment was in the same range as reported from earlier assessments in the area (Gullström et al., 2018) and similar in all vegetated subplots, and did not reflect either the relative cover of calcareous algae or the CaCO_3_ production rate. When degraded, *Halimeda* carbonate disintegrates into thin flakes that are easily transported with currents over the meadows (Fornosa et al., 1992; Prager et al., 1996). This transport of CaCO_3_ could possibly explain why all the vegetated subplots had similar inorganic carbon load, but since the unvegetated sediments had drastically lower levels of inorganic carbon it could not be the only explanation. One reason for this can be that unvegetated sediment obviously lack the structural complexity for efficiently trapping sediment particles. Another explanation might be that there are many other CaCO_3_ producers in tropical seagrass meadows. In Chwaka Bay, the different *Halimeda* species have been shown to be the most important carbonate producers (Muzuka et al., 2001), but there are many reports of other seagrass meadow inhabitants that are producing large amounts of CaCO_3_. For instance, in other tropical areas the epiphytic algal communities of different *Thalassia* species have been found to be major CaCO_3_ producers (Frankovich & Zieman, 1994; Land, 1970; Nelsen & Ginsburg, 1986) and the seagrass itself (*Thalassia testudinum*) has been reported to precipitate aragonite needles in their cell walls, and as external deposits on their leaves (Enríquez & Schubert, 2014). These contributions could provide equal levels of CaCO_3_ as the *Halimeda* species, thereby potentially equaling the inorganic carbon content between subplots, while the unvegetated sediment would receive less.

In summary, we found that sedimentary blue carbon storage levels were higher in mixed plant communities than when plants were growing in monospecific stands. This corresponded well with the total net areal biomass productivity measurements, where a mixture of seagrass and calcareous algae revealed the highest productivity levels. The findings clearly show that plant community composition could influence both ecosystem productivity and blue carbon sequestration in tropical seagrass meadows. The presence of calcareous algae within seagrass meadows should hence be carefully considered in conservation planning.

## CONFLICT OF INTEREST

The authors declare no competing interests.

## AUTHOR CONTRIBUTIONS

**Olivia J. Kalokora:** Conceptualization (equal); Data curation (equal); Formal analysis (equal); Investigation (lead); Methodology (lead); Project administration (equal); Validation (equal); Visualization (equal); Writing – original draft (equal); Writing – review & editing (equal). **Martin Gullstrom:** Conceptualization (equal); Data curation (equal); Formal analysis (equal); Funding acquisition (equal); Investigation (equal); Methodology (equal); Project administration (equal); Resources (equal); Supervision (equal); Validation (lead); Visualization (equal); Writing – original draft (equal); Writing – review & editing (equal). **Amelia S. Buriyo:** Funding acquisition (equal); Investigation (supporting); Methodology (supporting); Project administration (equal); Supervision (equal); Validation (equal); Writing – review & editing (equal). **Matern S. P. Mtolera:** Conceptualization (supporting); Funding acquisition (lead); Investigation (supporting); Methodology (supporting); Project administration (lead); Supervision (equal); Writing – review & editing (equal). **Mats Björk:** Conceptualization (equal); Data curation (equal); Formal analysis (equal); Funding acquisition (equal); Investigation (supporting); Methodology (supporting); Project administration (equal); Supervision (equal); Validation (equal); Visualization (equal); Writing – original draft (equal); Writing – review & editing (equal).

## Supporting information

Appendix S1

## Data Availability

The data that support the findings of this study are openly available in Dryad at https://doi.org/10.5061/dryad.sxksn0350.
