# Supplementary material for: Seagrass meadows mixed with calcareous algae have higher plant productivity and sedimentary blue carbon storage
Source: Ecol Evol. 2022 Feb 14;12(2):e8579. doi: 10.1002/ece3.8579 (PMC8843821; doi:10.1002/ece3.8579)
Supplement: Supplementary file 1 — Appendix S1 [file ECE3-12-e8579-s001.docx]

**Supplementary material:**

**Table S1.** *T. hemprichii* and *H. opuntia* average density, height and biomass measured in the three sites. Abbreviations are AGB above ground biomass and BGB belowground biomass. Data are Mean ± SE, n=9.

| Characteristics | Species | Chwaka | Mkanjani | Marumbi |
| --- | --- | --- | --- | --- |
| Shoot density (m^-2^) | *T. hemprichii* | 350 ± 57.7 | 403 ± 56.9 | 508 ± 46.1 |
| Canopy height (cm) | *T. hemprichii* | 13.2 ± 0.2 | 14.0 ± 0.4 | 15.8 ± 0.4 |
| AGB (g DW m^-2^) | *T. hemprichii* | 308 ± 16.7 | 359 ± 25.6 | 506 ± 33.5 |
| BGB (g DW m^-2^) | *T. hemprichii* | 333 ± 11.2 | 458 ± 27.9 | 538 ± 26.6 |
| Thalli density (m^-2^) | *H. opuntia* | 61 ± 8.3 | 58 ± 11.7 | 73 ± 11.8 |
| Canopy height (cm) | *H. opuntia* | 7.5 ± 0.2 | 7.2 ± 0.2 | 6.8 ± 0.1 |
| AGB (g DW m^-2^) | *H. opuntia* | 417 ± 21.1 | 497 ± 16.6 | 579 ± 17.4 |

**Table S2.** Physicochemical parameters at the three different sites. Data are Mean ± SE, n=9

| Water parameter | Chwaka | Mkanjani | Marumbi |
| --- | --- | --- | --- |
| Temperature (°C) | 32.50±0.46 | 31.18±0.36 | 30.24±0.48 |
| Light (µmol photons m^-2^s^-1^) | 982.77±42.39 | 927.31±78.45 | 562.93±97.31 |
| Salinity | 30.34±0.17 | 30.96±0.07 | 31.19±0.21 |
| pH | 8.27±0.02 | 8.24±0.03 | 8.17±0.03 |
| DO (mgL^-1^) | 9.78±0.21 | 8.64±0.21 | 8.85±0.18 |

**Table S3**. Plant (seagrass and calcareous [calc] algae) cover, biomass and productivity as well as CaCO3 productivity and content (mean ± SE) measured in the different subplots (n = 9). Letters in red colour indicate significant differences (based on results from Tukey´s post hoc tests) between different subplots for each response variable. * = response variables where data have been transformed due to heterogenous variances (for details, see Data analysis).
